# Primary Malignant Melanoma of the Nasolacrimal Duct Presenting Without Hemolacria: A Case Report and Literature Review

**DOI:** 10.3390/curroncol33020117

**Published:** 2026-02-17

**Authors:** Won Gun Kwack, Hong Jun Kim

**Affiliations:** 1 Department of Pulmonary, Allergy, and Critical Care Medicine, College of Medicine, Kyung Hee University Hospital, 23 Kyung Hee Dae-ro, Dongdaemun-gu, Seoul 02447, Republic of Korea; wongunnim@khu.ac.kr; 2 Department of Medical Oncology, College of Medicine, Kyung Hee University Hospital, 23 Kyung Hee Dae-ro, Dongdaemun-gu, Seoul 02447, Republic of Korea

**Keywords:** nasolacrimal duct melanoma, lacrimal sac tumor, mucosal melanoma, adjuvant radiotherapy, hemolacria

## Abstract

Primary malignant melanoma of the nasolacrimal duct is a rare and aggressive mucosal neoplasm. Its diagnosis is frequently delayed because early symptoms often mimic benign inflammatory conditions like chronic dacryocystitis. While hemolacria is considered a hallmark sign, it is not universally present. We present a case of a 78-year-old woman with a 1-year history of persistent epiphora and discharge from the left eye, without hemolacria. Initial lacrimal irrigation suggested obstruction. Computed tomography (CT) revealed a soft tissue lesion with widening of the lacrimal bony canal but without gross destruction. An incisional biopsy confirmed malignant melanoma. The patient underwent wide surgical resection, including medial maxillectomy, followed by adjuvant intensity-modulated radiotherapy (50 Gy). The tumor was BRAF wild-type. At 24 months post-treatment, the patient remains disease-free with no evidence of recurrence or metastasis. This case highlights that the absence of hemolacria should not preclude the suspicion of malignancy in refractory lacrimal obstruction. Early cross-sectional imaging and biopsy are essential for accurate diagnosis. Furthermore, this case suggests that a multimodal approach combining wide surgical excision and adjuvant radiotherapy may contribute to durable local control in selected patients.

## Simple Summary

Nasolacrimal duct melanoma is an extremely rare and aggressive cancer affecting the tear drainage system. Because its early symptoms—such as tearing and eye discharge—look exactly like common benign infections, doctors often miss the diagnosis until the tumor has grown large. It is widely believed that “bloody tears” are a key warning sign of this cancer. However, we report a case of an elderly woman who had this cancer but never experienced bloody tears; she only had persistent tearing and discharge. By using advanced imaging and biopsy early, we diagnosed the cancer before it spread. She was treated with a combination of surgery and radiation therapy and remains cancer-free after two years. This report is important because it warns doctors not to wait for bloody tears to suspect cancer. It suggests that early diagnosis followed by complete surgical resection, with consideration of adjuvant radiotherapy, may provide durable local control in selected patients.

## 1. Introduction

Primary malignant melanoma of the nasolacrimal duct is a rare and aggressive neoplasm. While melanomas of the head and neck region are uncommon, those arising specifically from the lacrimal sac or nasolacrimal duct represent a distinct clinical entity, accounting for approximately 0.7% of all ocular melanomas and less than 5% of lacrimal sac tumors [1,2]. Compared to sinonasal or oral mucosal melanomas, which together account for over 80% of head and neck mucosal cases, primary nasolacrimal duct melanoma is exceptionally rare and poses unique diagnostic challenges due to its occult anatomical location [1]. Unlike cutaneous melanoma, mucosal melanoma in this region exhibits unique biological behavior characterized by locoregional aggressiveness and a propensity for recurrence due to the complex anatomy of the nasolacrimal system, which facilitates occult invasion into surrounding bony structures and paranasal sinuses [3]. Biologically, these tumors are distinct from their cutaneous counterparts, exhibiting a significantly lower frequency of BRAF mutations (<6% in mucosal vs. ~50% in cutaneous) and a higher prevalence of KIT or NRAS alterations (approximately 10–25% each) [1]. Although the pathogenesis remains not fully elucidated, it is hypothesized that these tumors originate from melanocytes that aberrantly migrated from the neural crest to the lacrimal sac epithelium or conjunctiva during embryogenesis [4]. However, it should be noted that this theory remains speculative, as definitive molecular or developmental confirmation of this migratory pathway in the human nasolacrimal system is currently lacking.

The prognosis of mucosal melanoma is generally poorer than that of cutaneous melanoma, largely due to late detection and the rich lymphatic and vascular supply of the mucosa, which promotes early metastasis. Clinical diagnosis of nasolacrimal duct melanoma presents significant challenges. The initial presentation is often non-specific, with symptoms such as epiphora and a palpable medial canthal mass that closely mimic benign inflammatory conditions like chronic dacryocystitis [5]. While hemolacria (bloody tears) has been historically emphasized as a hallmark sign suggestive of malignancy in the lacrimal system, recent systematic reviews indicate that it is not universally present [6]. Consequently, diagnosis is frequently delayed, with many patients undergoing conservative treatment for presumed inflammation or being diagnosed incidentally during dacryocystorhinostomy (DCR) [7].

Herein, we present a case of primary malignant melanoma of the nasolacrimal duct in a patient with a 1-year history of persistent discharge without hemolacria. The patient was successfully managed with wide surgical resection followed by adjuvant radiotherapy, achieving disease-free survival at 24 months. We discuss the diagnostic pitfalls associated with non-specific presentations, the differential diagnosis from other lacrimal system pathologies, and highlight the potential efficacy of adjuvant radiotherapy in achieving local control. This report emphasizes the critical role of a multidisciplinary approach involving ophthalmology, surgical oncology, and radiation oncology in the management of this rare malignancy.

## 2. Case Presentation

**Figure 1:**
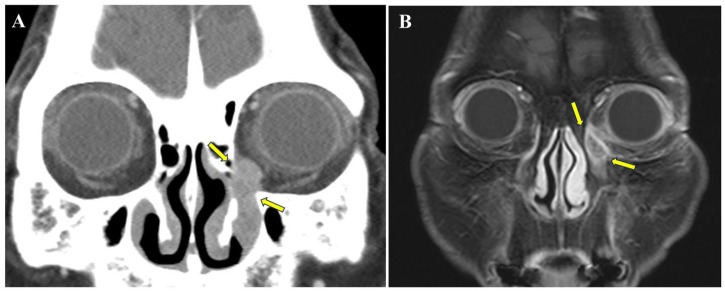
Preoperative imaging findings of the nasolacrimal duct melanoma. (**A**) Contrast-enhanced coronal orbital computed tomography (CT) scan in soft tissue window reveals an elongated, homogenously enhancing tumor lesion (yellow arrows) occupying the left medial canthal region and extending into the nasolacrimal duct. Note the widening of the lacrimal bony canal without gross bony destruction. (**B**) Coronal T1-weighted fat-suppressed contrast-enhanced magnetic resonance imaging (MRI) of the head and neck demonstrates a heterogeneously enhancing tumor lesion (yellow arrows) involving the left medial canthus and the upper portion of the nasolacrimal duct, corresponding to the CT findings.

A 78-year-old woman presented with a 1-year history of persistent discharge from the left eye, accompanied by a firm and uncomfortable sensation at the medial canthal area. She had no history of chronic liver disease, tuberculosis, diabetes mellitus, or other systemic illnesses, and her only medication was an antihypertensive agent. There was no prior history of hospitalization or surgery. At presentation, she reported persistent epiphora and mucopurulent discharge without hemolacria. On ophthalmologic examination, best-corrected visual acuity was 0.4 in the right eye and 0.6 in the left eye, and intraocular pressure was 16 and 17 mmHg, respectively. Extraocular movements were full, pupils were reactive to light, and no relative afferent pupillary defect was observed. Abundant discharge was noted from the left conjunctiva. Lacrimal irrigation demonstrated good passage in the right eye, whereas the left eye showed reflux with delayed minimal passage. Based on these findings, a tumorous lesion involving the lacrimal sac or nasolacrimal duct was suspected, and a contrast-enhanced orbital computed tomography (CT) scan was performed (Figure 1A). Imaging revealed an elongated soft tissue lesion, measuring approximately 2.1 cm × 0.9 cm (long and short axis, respectively), occupying the left medial canthal region and involving the nasolacrimal apparatus, with homogeneous enhancement and widening of the lacrimal bony canal, without evidence of perilesional infiltration or bony destruction. The radiologic differential diagnosis included an epithelial tumor such as papilloma or squamous cell carcinoma, lymphoma, and less likely inflammatory conditions such as dacryocystitis, and tissue confirmation was recommended. Subsequently, an incisional biopsy was performed under local anesthesia. Histopathological examination revealed malignant melanoma.

Following histologic confirmation, additional imaging studies were performed as part of a preoperative evaluation for curative-intent surgery. Contrast-enhanced magnetic resonance imaging (MRI) of the head and neck demonstrated a heterogeneously enhancing soft tissue mass involving the left medial canthus and upper nasolacrimal duct, without evidence of additional abnormal soft tissue lesions within the scanned field (Figure 1B). For systemic staging, 18F-fluorodeoxyglucose positron emission tomography–computed tomography (PET–CT) revealed no evidence of regional or distant metastasis. According to the American Joint Committee on Cancer 8th edition Tumor–Node–Metastasis staging system for head and neck mucosal melanoma, the clinical stage was classified as cT3N0M0 (Stage III). Preoperative nasal endoscopy was not performed; however, the patient reported no nasal symptoms (e.g., epistaxis or nasal obstruction), and cross-sectional imaging (CT and MRI) did not suggest an additional sinonasal mucosal lesion.

**Figure 2:**
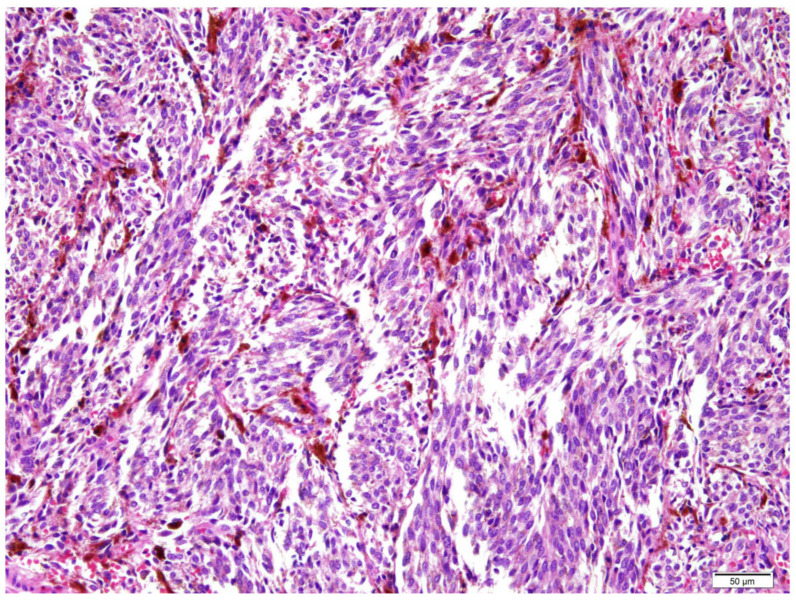
Histopathological findings of the resected nasolacrimal duct tumor. High-power photomicrograph demonstrates a dense proliferation of neoplastic cells arranged in a fascicular pattern. The tumor cells exhibit spindle-shaped morphology with hyperchromatic, pleomorphic nuclei. Prominent intracellular and extracellular brown melanin pigment deposition is evident throughout the stroma, confirming the diagnosis of malignant melanoma (Hematoxylin and Eosin stain; scale bar = 50 µm).

The patient subsequently underwent definitive surgical resection under general anesthesia. The surgical approach involved a lateral rhinotomy incision to expose the lacrimal fossa. En bloc resection was performed, including the left nasolacrimal duct mass, the lacrimal sac, and a medial maxillectomy to ensure adequate clearance of the bony canal. The periorbita was preserved as there was no gross invasion into the orbital fat. Final histopathological examination of the surgical specimen (nasal cavity, left nasolacrimal duct, medial maxillectomy) confirmed malignant melanoma, measuring 2.2 × 1.2 × 0.6 cm, with a mitotic rate of 5 per 10 high-power fields (Figure 2). Immunohistochemical analysis was performed to confirm the diagnosis, revealing strong and diffuse positivity for specific melanocytic markers, including S-100 protein, HMB-45, and Melan-A. Conversely, the tumor was negative for epithelial markers (pan-cytokeratin), thereby supporting the diagnosis of melanoma and excluding epithelial malignancies such as squamous cell carcinoma. Tumor-infiltrating lymphocytes were absent, and microsatellites were not identified. All resection margins were free of tumor involvement, with the closest margins measuring 0.1 cm superiorly, 0.5 cm anteriorly, and 0.1 cm inferiorly. All surgical margins were inked and evaluated on permanent sections, and the closest margin was determined by microscopic measurement of the minimum distance from invasive tumor to the inked resection surface. There was no evidence of lymphatic, small vessel, neural, or perineural invasion. Postoperatively, the patient experienced mild impairment of medial eyelid closure accompanied by epiphora, attributable to resection of the medial canthal region; however, no visual functional deficits, including diplopia or reduction in visual acuity, were observed.

Given the rarity of primary nasolacrimal duct melanoma and the absence of established treatment guidelines, the patient’s management plan was determined through a multidisciplinary discussion. In view of the anatomically constrained nasolacrimal duct/medial canthal subsite and the very close but negative surgical margins (closest superior and inferior margins, 0.1 cm each), adjuvant radiotherapy was recommended to reduce the risk of microscopic residual disease and improve local control. The patient’s good performance status and uncomplicated postoperative course supported the feasibility of postoperative radiotherapy. Systemic treatment was not pursued, owing to the limited supporting evidence in the adjuvant setting. The tumor was confirmed to be BRAF wild type, on BRAF V600 hotspot mutation testing (exon 15) performed on formalin-fixed paraffin-embedded biopsy tissue using a real-time PCR–based assay (PNAClamp™ BRAF Mutation Detection Kit; Panagene Inc., Daejeon, Republic of Korea); no V600E/D/K/R/A mutation was detected. If unresectable recurrence or distant metastasis occurs, immune checkpoint inhibitor therapy (e.g., anti–PD-1 therapy) would be considered based on guideline recommendations. Adjuvant radiotherapy was delivered using a linear accelerator-based intensity-modulated radiotherapy (IMRT) technique to improve local control. The clinical target volume (CTV) encompassed the postoperative tumor bed along the left nasolacrimal duct trajectory and the adjacent medial canthal surgical bed considered at risk for microscopic disease, while respecting nearby anatomic barriers. The planning target volume (PTV) received a total dose of 50 Gy in daily fractions of 2 Gy. An adaptive re-planning was performed during the treatment course to account for postoperative anatomic changes and to maintain target coverage while respecting organs at risk doses; on the final dose-volume histogram, PTV Dmean, Dmin, and Dmax (mean, minimum, and maximum dose, respectively) were 50.7 Gy, 47.5 Gy, and 54.8 Gy. The ipsilateral optic nerve Dmax was 40.6 Gy and the optic chiasm Dmax was 15.3 Gy; the ipsilateral lens Dmax was 46.9 Gy. The patient completed radiotherapy without acute toxicities of grade ≥2 according to CTCAE v5.0; at the 24-month follow-up, there were no late grade ≥2 ocular toxicities, including decreased visual acuity, diplopia, keratitis, cataract or optic neuropathy. At the most recent follow-up, 24 months after completion of treatment, the patient remains alive with no evidence of local recurrence or distant metastasis. A detailed case timeline, including diagnostic work-up, treatment, and follow-up assessments, is summarized in Table 1.

## 3. Discussion

This case illustrates the diagnostic and therapeutic challenges associated with primary malignant melanoma of the nasolacrimal duct, a rare mucosal melanoma that frequently presents with non-specific symptoms and is therefore prone to delayed diagnosis. In the present patient, persistent epiphora and medial canthal discomfort without hemolacria initially mimicked benign inflammatory disease, a pattern that has been consistently reported in previous studies of lacrimal drainage system malignancies [2,5,7]. Despite these diagnostic challenges, timely tissue confirmation followed by comprehensive preoperative imaging enabled curative-intent surgical resection with negative margins. The subsequent use of adjuvant radiotherapy resulted in durable local control, with no evidence of disease at 24 months of follow-up. This case is clinically relevant because it highlights a common diagnostic pitfall in routine ophthalmologic practice and suggests that a multimodal treatment approach may achieve favorable outcomes even in this anatomically complex and biologically aggressive malignancy.

To provide a comprehensive context for this rare entity, we conducted a systematic literature search using the PubMed and Google Scholar databases up to December 2025. The search terms included “nasolacrimal duct melanoma,” “lacrimal sac melanoma,” and “lacrimal drainage system melanoma.” We prioritized reports describing primary melanomas arising specifically from or extensively involving the nasolacrimal duct. We excluded cases of secondary metastasis or those with insufficient clinical data. A summary of representative cases from the literature, focusing on presenting symptoms (specifically hemolacria), treatment modalities, and outcomes, is presented in Table 2.

The non-specific clinical presentation of nasolacrimal duct melanoma remains a major contributor to delayed diagnosis and initial mismanagement. Symptoms such as epiphora, mucopurulent discharge, and medial canthal fullness are common in routine ophthalmologic practice and are frequently attributed to benign conditions, including chronic dacryocystitis or primary nasolacrimal duct obstruction [2,5,7]. Importantly, the absence of hemolacria—traditionally regarded as a warning sign of malignancy—does not exclude an underlying neoplastic process, as demonstrated in both prior reports and the present case [2,6,12]. Although fewer than 50 cases of primary nasolacrimal duct melanoma have been reported, a recent systematic review indicates that approximately 43.5% of nasolacrimal duct melanoma cases present without hemolacria, suggesting that this clinical presentation is not uncommon within this rare entity [13]. Therefore, clinicians should maintain a high index of suspicion when nasolacrimal symptoms are persistent, progressive, or refractory to conservative treatment. Specifically, we recommend early cross-sectional imaging (CT and/or MRI) in cases of persistent epiphora to rule out mass lesions or bony erosion. In the differential diagnosis, other malignancies such as squamous cell carcinoma, which is the most common lacrimal sac malignancy, transitional cell carcinoma, and lymphoma must be considered. While lymphoma often presents as a painless, rubbery mass, carcinomas and melanomas tend to be firmer and may cause more structural destruction. In such situations, we advocate for a low threshold for biopsy, either preoperatively or intraoperatively during DCR, particularly when atypical clinical or radiologic features are identified. Excluding malignancy before proceeding with routine DCR is crucial to avoid delay in definitive diagnosis and potential tumor dissemination [7,12].

Imaging plays a pivotal role in both diagnosis and treatment planning. CT is useful for detecting soft tissue masses and identifying subtle findings such as widening of the lacrimal bony canal, which may suggest a space-occupying lesion rather than simple inflammatory disease [3,14]. In our case, the widening of the bony canal was a critical clue distinguishing the lesion from simple dacryocystitis. While frank bony destruction is often associated with aggressive infiltration, bony remodeling or widening without overt osteolysis—as observed in this patient—has been described in lacrimal sac malignancies and may suggest a relatively contained process, although this finding alone does not definitively rule out microscopic invasion [14]. MRI provides superior delineation of soft tissue extent and potential invasion into adjacent structures, thereby facilitating accurate assessment of local disease [15]. In addition, PET–CT contributes to systemic staging and supports surgical decision-making by excluding regional or distant metastasis [2,7,15]. In the present case, the combined use of CT, MRI, and PET–CT enabled timely diagnosis and appropriate selection of a curative-intent treatment strategy, underscoring the importance of comprehensive imaging in patients with atypical or persistent nasolacrimal symptoms.

Surgical resection remains the cornerstone of management for nasolacrimal duct melanoma, with the primary objective being en bloc removal of the tumor with clear margins. However, achieving adequate negative margins is frequently complicated by the intricate anatomy of the medial canthus and the proximity to critical structures such as the orbit, skull base, and paranasal sinuses. Due to these anatomical constraints, local recurrence rates following surgery alone are historically high, reported to reach up to 50% in some series [16]. The management of regional lymph nodes remains controversial. While elective neck dissection is not routinely recommended for clinically N0 mucosal melanoma due to the lack of proven survival benefit, some authors advocate for sentinel lymph node biopsy. In our case, given the negative PET-CT findings and the lack of palpable adenopathy, we opted for observation of the neck. To mitigate the risk of local recurrence, adjuvant radiotherapy is increasingly utilized as a complementary modality. Although melanoma has traditionally been regarded as a radio-resistant tumor, accumulating evidence in head and neck mucosal melanoma suggests that postoperative radiotherapy significantly improves locoregional control rates compared to surgery alone [11,17]. While its impact on overall survival remains controversial, the ability of adjuvant RT to delay or prevent local failure is paramount in this anatomically critical region, where recurrence often necessitates disfiguring salvage surgery (e.g., orbital exenteration). Therefore, maximizing local control is particularly valuable for preserving visual function and quality of life [18]. In the present case, although all margins were negative, the closest superior and inferior margins were only 0.1 cm, and complete anatomic clearance of the nasolacrimal duct within the lacrimal bony canal is intrinsically limited; therefore, adjuvant IMRT was selected to mitigate the risk of occult residual disease and subsequent local recurrence. Regarding dose selection, postoperative IMRT to 50 Gy in 25 fractions was chosen as a conventional adjuvant regimen for presumed microscopic disease after complete resection, balancing the intent of local control with the need to spare adjacent critical ocular structures in this anatomically constrained region [18]. The sustained disease-free status at 24 months in our patient supports the utility of this multimodal approach and aligns with recent recommendations advocating for adjuvant radiation in cases where wide anatomical clearance is challenging.

The role of systemic therapy in the adjuvant management of localized nasolacrimal duct melanoma remains undefined due to the scarcity of high-level evidence. Unlike cutaneous melanoma, mucosal melanomas of the head and neck frequently lack BRAF mutations, as observed in our patient who was BRAF wild type, thereby limiting the utility of BRAF-targeted agents [19]. Instead, mucosal melanomas show a higher frequency of c-KIT aberrations and NRAS mutations [20]. Although we did not test for c-KIT or NRAS in this patient due to the localized nature of the disease, genetic profiling could offer therapeutic avenues in the setting of recurrence or metastasis. While immune checkpoint inhibitors (e.g., anti-PD-1 agents such as pembrolizumab or nivolumab) have revolutionized the treatment of advanced melanoma, their efficacy in the adjuvant setting for non-metastatic mucosal subtypes is less established compared to cutaneous counterparts [21,22]. Although recent trials are investigating adjuvant immunotherapy in high-risk mucosal melanoma, current guidelines generally reserve systemic immunotherapy for unresectable locoregional recurrences or distant metastases [22]. Similarly, the role of neoadjuvant therapy, while showing promise in resectable cutaneous melanoma, remains experimental in mucosal subtypes and is not currently recommended outside of clinical trials. Consequently, our multidisciplinary team prioritized local control through surgery and radiotherapy, withholding systemic treatment until potential disease progression. Given the rarity of this malignancy, the execution of large-scale randomized clinical trials is inherently difficult. Therefore, the continued accumulation of clinical data through multi-institutional registries is imperative to establish standardized treatment protocols and define the optimal integration of surgery, radiotherapy, and emerging systemic agents for this orphan disease [20].

## 4. Conclusions

Primary malignant melanoma of the nasolacrimal duct is a diagnostically challenging entity that frequently mimics benign inflammatory diseases. This case underscores a critical clinical lesson: the absence of hemolacria should not lower the index of suspicion. Clinicians must consider malignancy in patients presenting with persistent epiphora or purulent discharge that is refractory to conventional management. Early utilization of cross-sectional imaging is paramount. In our patient, the specific radiologic finding of lacrimal bony canal widening without gross osseous destruction suggested a potentially localized stage of invasion. In this case, the absence of extensive bony involvement was consistent with the feasibility of complete surgical resection, which was followed by a favorable clinical outcome. Furthermore, this report highlights the feasibility and potential role of a multimodal, curative-intent strategy in selected patients. Despite the limited systemic options associated with BRAF wild-type status, the patient remained disease-free at 24 months after wide surgical excision and adjuvant radiotherapy. Ultimately, successful outcomes depend on early diagnosis driven by a high index of suspicion and aggressive locoregional control. This case reinforces the critical need for a multidisciplinary approach—integrating ophthalmology, surgical oncology, and radiation oncology—to ensure diagnostic accuracy and optimize treatment sequencing in this rare and potentially lethal malignancy.

## Figures and Tables

**Table 1:** Case timeline.

Timepoint	Key Events	Findings/Management
Month −12	Symptom onset	Persistent epiphora and discharge (no hemolacria).
Month 0	Initial ophthalmologic evaluation	Irrigation: right patent; left reflux with delayed minimal passage.
Month 0	Orbital CT	Left medial canthal/nasolacrimal apparatus mass; widening of lacrimal bony canal; no bony destruction.
Month 0	Incisional biopsy	Malignant melanoma.
Month 0–1	Preoperative staging	MRI: localized lesion; PET–CT: no regional/distant metastasis.
Month 1	Definitive surgery	En bloc resection with medial maxillectomy; margins negative (closest 0.1 cm).
Month 2	Multidisciplinary discussion	Adjuvant RT recommended (anatomic constraint, very close margins).
Month 2–3	Adjuvant RT	IMRT 50 Gy in 25 fractions; completed without significant acute toxicity.
Month 24	Follow-up	No evidence of local recurrence or distant metastasis.

Abbreviations: CT, computed tomography; MRI, magnetic resonance imaging; PET–CT, positron emission tomography–computed tomography; RT, radiotherapy; IMRT, intensity-modulated radiotherapy; hemolacria, bloody tears.

**Table 2:** Summary of reported cases of primary malignant melanoma involving the nasolacrimal duct.

Study (Year)	Age/Sex	Primary Site	Hemolacria	Treatment	Outcome
Lewis et al. (2006) [8]	77/F	NLD	No	Surgery (wide excision)	DOD (18 mo)
Esteban et al. (2007) [9]	75/F	NLD	Yes	Surgery + RT	NED (24 mo)
Chou et al. (2010) [10]	78/M	NLD	No	Surgery (limited)	DOD (5 mo)
Pujari et al. (2014) [6]	48/M	LS & NLD	No	Surgery + CTx	AWD (12 mo)
Terazawa et al. (2025) [11]	70/F	NLD	Yes	Surgery + RT + ICI	NED (12 mo)
Present Case (2026)	78/F	NLD	No	Surgery + RT	NED (24 mo)

Abbreviations: F, female; M, male; NLD, nasolacrimal duct; LS, lacrimal sac; RT, radiotherapy; CTx, chemotherapy; ICI, immune checkpoint inhibitor; DOD, died of disease; NED, no evidence of disease; AWD, alive with disease; mo, months.

## Data Availability

The data presented in this study are available in this article.

## Abbreviations

The following abbreviations are used in this manuscript:

CT	Computed Tomography
CTV	Clinical Target Volume
DCR	Dacryocystorhinostomy
IMRT	Intensity Modulated Radiotherapy
MRI	Magnetic Resonance Imaging
PET-CT	Positron Emission Tomography–Computed Tomography
PTV	Planning Target Volume
